# Preparation and Characterization of Self-Healing Mortar Based on “Build-In” Carbonation

**DOI:** 10.3390/ma13030644

**Published:** 2020-02-01

**Authors:** Xin Wang, Wenting Li, Zhengwu Jiang

**Affiliations:** Key Laboratory of Advanced Civil Engineering Materials Ministry of Education, Tongji University, Shanghai 200092, China; 1830628@tongji.edu.cn (X.W.); jzhw@tongji.edu.cn (Z.J.)

**Keywords:** self-healing, cement-based, compatibility, Na_2_CO_3_, CaCO_3_, microstructural test

## Abstract

In this study, a new type of cement-based healing pellets (CHPs) were proposed to accelerate the healing efficiency of concrete, which was mainly based on the introduced Na_2_CO_3_ on promoting the formation of calcium carbonate (CaCO_3_) in cracks. The effects of Na_2_CO_3_ on the characteristics of CHPs were firstly investigated, and then the properties of cement mortar mixed with CHPs were studied quantitatively, including the workability, mechanical properties and healing ability. The results showed that higher dosages of Na_2_CO_3_ in CHPs decreased the size range of pellets and reduced the setting time, fluidity and heat of hydration of mortar. Still more, CHPs reduced the early strength of mortar but kept the intensity growth rate stable such that it had nearly no negative effect on the later strength. With the content of CHPs increasing, the strength of mortar showed a decreasing trend, while the pore-filling efficiency and strength healing rate of mortar were further improved. In addition, as a new type of self-healing pellets for concrete based on the “build-in” carbonation, CHPs improved the strength and healing effectiveness of cement mortar. When the dosage of Na_2_CO_3_ in CHPs and the content of CHPs in mortar were at 10% and 25%, respectively, mortar obtained highest strength in the later stage and the best healing effect.

## 1. Introduction

As an irreplaceable construction material, concrete is confronted with a potential threat to the durability after cracked [1]. Autogenous or autonomous healing of fine cracks has been reported in many studies, and can be defined as the ability of materials to actively transform energy and matter from the surrounding to heal the damage [2].

Autogenous healing of cracks in concrete bridges was investigated in the 1930s [3,4], which mainly relied on the continued hydration and carbonization of cement mortar to fill cracks with hydrated gels and calcium carbonate crystals [5,6,7,8,9,10]. The carbonation process was proven to be an efficient way to heal the cracks by Edvardsen [11]. It is noteworthy that the formation of calcite in this process was limited by the concentration of CO_2_ and the compactness of samples, the filling and repairing effect of which can only be maintained on the surface of the crack in the natural state [12]. In order to improve the self-healing ability of cement structure, a common way is introducing a repairing substance into the mortar [6]. This way is called autonomic healing and has got lots of attention in recent years. Some scholars have made great achievements by introducing microorganisms, shape memory alloys, microcapsules or hollow glass fibers into concrete to accelerate the repair process. Once the concrete structures are damaged, these repair materials would be aroused to heal the cracks and improve the mechanical properties.

However, the incompatibility between the repair materials and cement mortar may cause other forms of damage, like strength loss and durability reduction of mortar [1,9,13]. Hence, some scholars adopted compatible strategies by imitating autogenous healing to heal cracks [1]. This method also mainly depends on the formation of hydration gel and calcium carbonate crystal in later stages in the water environment, which could retain the compatibility of cement mortar and healing materials. The effects of mineral admixtures including an expansive agent, geo-materials and chemical agents on the self-healing properties of mortar were studied by Kishi et al. [14,15,16]. On this basis, the active silica and crystal admixtures were studied by Sisomphonl et al. [17]. Jiang et al. [18] investigated the healing effect of minerals and found that high pHs and high temperatures accelerated the healing progress. Nonetheless, the issues on the long-term stability and repeatability of the healing ability have not yet been properly addressed.

Li et al. [19] coated ceramists with waterproofing material or Portland cement paste, respectively. It was found that the utilization of Portland cement paste as a coating shell improved the compatibility of mortar and ceramists better than waterproofing material. Wang et al. [20] chose ceramists as the carrier to safeguard the healing substance, which made up with expansive agents, crystalline admixture and calcium hydrogen phosphate. The results proved that taking ceramists as a container to design repeatable self-healing concrete was a feasible way. The pan-pelletizing technology was utilized to prepare mineral pellets by Alghamri [21,22], then those prepared pellets were coated by PVA to reduce the invalid release of mineral. These pellets showed a remarkable performance on crack sealing but forfeited the expected mechanical properties and strength healing rates of mortar. Choi [23,24] et al. introduced ultra-fine CO_2_ bubbles into the saturated Ca(OH)_2_ solution to promote the formation of CaCO_3_; it was found that the crystal forms of CaCO_3_ could be controlled by optimizing the temperature and pH, but there was no further study on the mechanical effect. Some scholars [15,25] introduced NaHCO_3_ and Na_2_CO_3_ into mortar to investigate the cementitious recrystallization with expansive agents. They affirmed that carbonates increased the self-healing ability of concrete, while the strength of mortar decreased in the alkalescent mix water. It has been proven that the morphology of the grains of coarse aggregate has an enormous effect on the compressive strength and stiffness of concretes; the proper incorporation of irregular aggregate into mortar improved their performance [26,27].

The previous studies showed that the introduced healing materials decreased the strength of cement mortar [28]. In order to prepare a new kind of self-healing pellets, which have better compatibility with concrete, cement-based self-healing pellets were designed and investigated in this study. CO_3_^2-^ was introduced into cement mortar through the pellets to realize the build-in carbonation and realize the build-in carbonation and improve the healing efficiency. The compatibility between the cement-based healing pellets (CHPs) and cement mortar was ensured without an organic shell around the pellets. The characteristics of CHPs met three merits: the compatibility of packaging shell and mortar; the shell is strong enough to protect repair agents while mixing; pellets exist stabilize in alkaline environments [29,30]. 

Then, a series of studies were carried out on those pellets. Firstly, the feasibility of this method was studied by the characteristics of CHPs, such as the contents of CO_3_^2-^ and its release rate in water. Next, the effects of CHPs on the fresh mortar were studied by the flowability, setting time and hydration heat. Then, the effects of pellets on the mechanical properties and healing rates of mortar were investigated by the test of compressive and four-point bending, combined with the acoustic emission (AE) test.

## 2. Experiments

### 2.1. Raw Materials 

**Figure 1:**
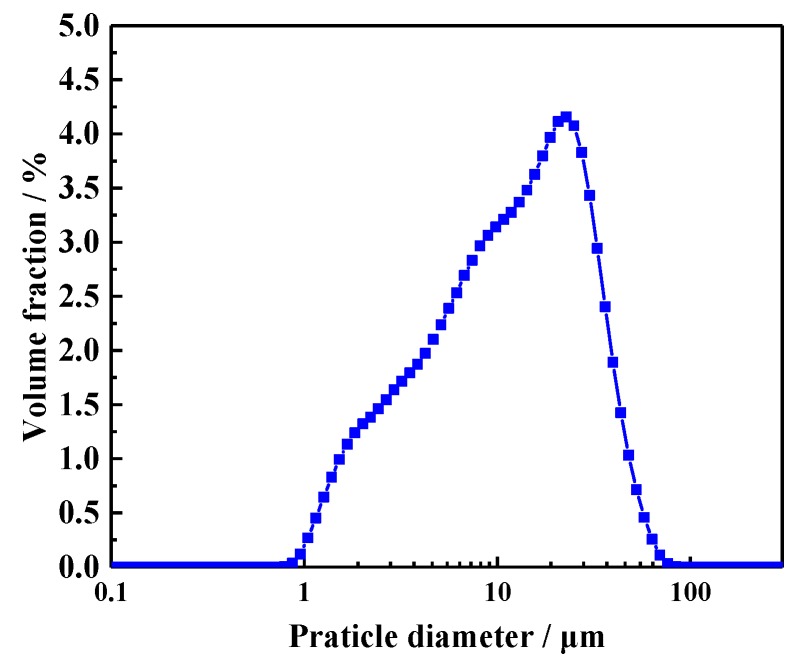
Particle size distribution of cement.

The cement used for the preparation of CHPs and mortar mixtures was Ordinary Portland Cement (CEM, I 52.5N, which was supplied by Jiangnan-xiaoyetian Cement Co., Ltd. and produced in Nanjing, Jiangsu, China.) with a particle density of 2.7–3.2 g/cm^3^. Its specific surface area range was 0.30–0.40 m^2^/g. The particle size distribution of the cement was measured by laser particle size analysis (LS320) (Beckman Coulter, Brea, CA, USA), as shown in Figure 1. Its average particle size was 16.1458 μm. The grain size of quartz sand ranged from 0.425 to 0.212 mm. Its specific gravity was 2.65 g/cm^3^. The chemical compositions are presented in Table 1. River sand was provided by a local supplier of building materials, with a maximum grain size of 3 mm and bulk density of 1.577 g/cm^3^.

The test of Na_2_CO_3_ was referenced to ASTM E359-17 [31]. It has a density of 0.88 g/mL, a total alkalinity is 99.63%, its percentage of loss on heating is 0.3%, the percentage of NaCl is 0.014% and the screen redundancy of 180 μm is 94%. 

### 2.2. Preparation and Test of CHPs

#### 2.2.1. Pelletization Process

The preparation of CHPs requires a low water-to-cement ratio (*w/c*), which is 0.22 in this paper. The dosages of Na_2_CO_3_ introduced into pellets were divided into 0%, 5%, 10% and 15%. The quartz sand was mixed to disperse pellets, which ensures that the diameter and shape of CHPs meet the experimental requirements. In this experiment, the content of quartz sand was prepared into pellets every 10 g from 0 to 100 g, and without its incorporation, Pellets A could not be formed. However, when the content of quartz sand was high, the diameter of Pellets D decreased obviously. When the mass of quartz sand was 30 g per group, the preparation of CHPs was the most beneficial for the preparation of pellets. Detailed mix proportions of CHPs are shown in Table 2. 

**Figure 2:**
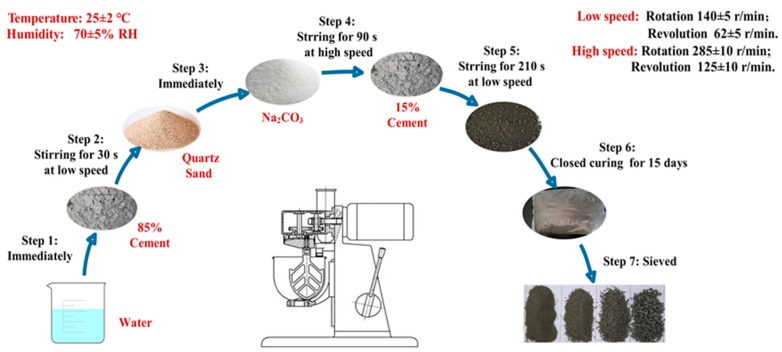
Pelletization process of CHPs.

The pelletization processes of CHPs were carried out in a JJ-5 cement mixer (which was produced in Shanghai, China and supplied by Shanghai Dongxing Building Materials Test Equipment Co., Ltd.) in an environment of 25 ± 2 °C and humidity of 70% ± 5% RH. The process of feeding in raw materials included seven steps as shown in Figure 2. At the first 30 s, 85% cement was mixed with water in the mixer at a low speed, then quartz sand and Na_2_CO_3_ were added in the cement paste. After mixing them for another 90 s at high speed, the remained 15% cement was added into the mixture and stirred for 210 s at low speed. The total preparation procedure was 4 min. Later, the mixture was sealed inside a clear plastic bag at 25 ± 2 °C to prevent the invasion of water and CO_3_^2-^ in the air. After curing for 15 days, the pellets were sieved into different size ranges.

#### 2.2.2. Size Grading and Bulk Density

**Figure 3:**
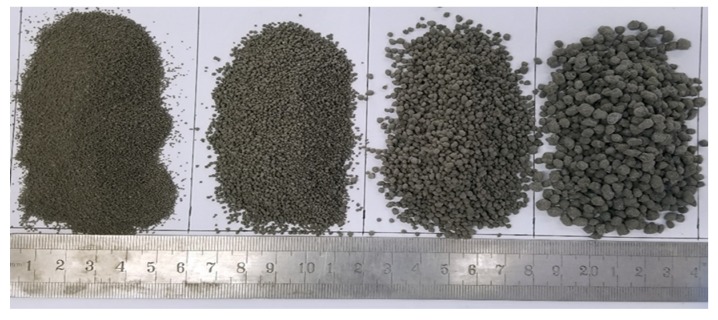
The appearance of CHPs.

The CHPs prepared with different dosages of Na_2_CO_3_ (See Figure 3) exhibited diverse grain size distributions. The pellets were sieved after 15 days of curing and divided into five size ranges, namely 0.16–0.315 mm, 0.315–0.63 mm, 0.63–1.25 mm, 1.25–2.5 mm and 2.5–5 mm. After baking for 48 h in an oven at 50 °C, the size grading and bulk density of the sieved pellets were tested according to JGJ-52-2006 [32].

#### 2.2.3. Microstructural Tests

In this study, X-ray diffraction analysis (XRD) and thermogravimetric analysis (TG) were used to quantitatively analyze the compositions of pellets and the content of unreacted Na_2_CO_3_. The specimens were dried, ground and passed through the 75 micron diameter sieve, which removed the interference of quartz sand. The apparatus used for XRD was a Rigaku Ultimate IV, which was made in Tokyo, Japan, and the diffractometer was operated at a scanning rate of 10 °/min in the scanning range 5°–90°. 

In the thermogravimetric analysis, the weights of samples A, B, C and D were 13.66 mg, 6.949 mg, 9.652 mg and 13.38 mg, respectively. The pyrolysis characteristics of CHPs were studied in the atmosphere of nitrogen by TA SDT Q600, which was produced by NETZSCH, Selb, Germany. The temperature range was set at 30–1000 °C, heating up at a rate of 10 °C /s. The mass fraction of CaCO_3_ in pellets (m_1_) was calculated by Equation (1), the mass fraction of Na_2_CO_3_ that had been consumed by CHPs (m_2_) is calculated by Equation (2) and the total mass fraction of Na_2_CO_3_ that was introduced in the pellets (m_3_) is given by Equation (3).

(1) $$m_{1}=w\times \frac{100.09}{44.0095}$$

(2) $$m_{2}=m_{1}\times \frac{105.99}{100.09}$$

(3) $$m_{3}=\frac{x}{1+0.22+x}$$

Then, the mass fraction of unreacted Na_2_CO_3_ (m_4_) was calculated by Equation (4):

(4) $$m_{4}=m_{3}-m_{2}$$

where w is the mass fraction of CO_2_, which was produced by the thermal decomposition of CaCO_3_, and x is the dosage of Na_2_CO_3_ that introduced into CHPs.

The surface characteristics and crystal forms of specimens were observed by an environmental scanning electron microscopy (ESEM, Quanta 200F) (FEI company, Hillsboro, OR, USA). The morphology of CHPs was recorded at magnifications of 1000× and 10,000× at a voltage of 15 KV. The samples used in the SEM observations were coated with platinum.

#### 2.2.4. The Ion Exchange Rate of CHPs

**Figure 4:**
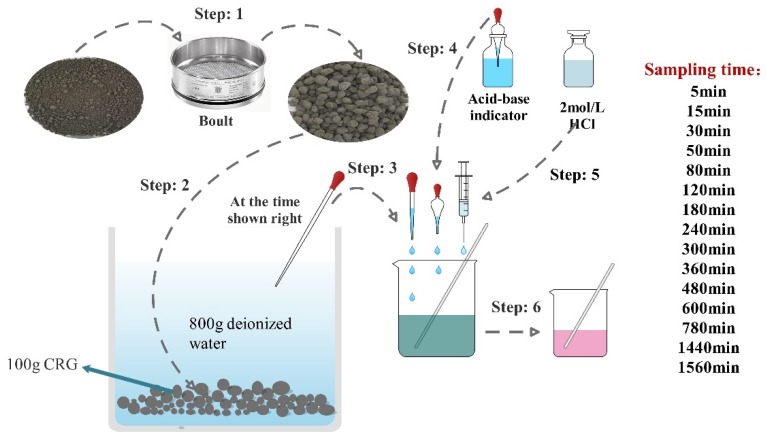
The flow chart of the titration test.

Due to the hydrophilicity of cement-based materials, Na_2_CO_3_ in the outer shell of CHPs is easy to dissolve in aqueous solution. In this study, the dissolving rate was measured by a titration experiment. The indicator was a mixture of bromocresol green and methyl red, and the standard titration solution was 1.5 mol/L hydrochloric acid. The experiment was carried out with 100 g dried CHPs and 800 g deionized water as shown in Figure 4. This timer was started when the pellets were put into the deionized water. Later, 10 g solution was taken out from the container by dropper at the sampling time and two drops of acid-base indicator was added into it. Then, hydrochloric acid was added into the solution drop by drop to observe the color change of the solution. The experiment was terminated when the color changed from green to pink. The dosage of hydrochloric acid and the concentration of alkali ions in the solution were collected.

### 2.3. Preparation and Test of Mortar

#### 2.3.1. Preparation of Mortar

In this study, the effects of CHPs on the properties of cement mortar were investigated on the base of Table 3. The types, sizes and contents of pellets were mainly taken into consideration. The mixing proportions were designated in a w/c ratio of 0.5 and cement–sand (c/s) ratio of 0.4. The contents of CHPs to the river sand of Group K were 10%, 25% and 40%. The specimens were prepared and cured according to GB/T 17671-1999 [33].

The pellets were divided into two groups of grain size, namely small pellets and large pellets. In the group of small pellets, the size ranges were mainly of 0.65–1.25 mm and 1.25–2.5 mm, which had a ratio of 2. The large pellets were in size ranges of 2.5–5 mm and 1.25–2.5 mm with a ratio of 2.

#### 2.3.2. Workability Test

##### Fluidity and Setting Time of Fresh Mortar

To determine the effect of CHPs on the fluidity of the fresh mortar, CHPs were introduced into the mortar as a partial replacement of sand as shown in Table 3. The fluidity was determined according to BS EN 1015–3:1999 [34]. The setting time of mortar was measured by a concrete penetration resistance tester based on JGJ/T 70-2009 [35]. A steel container with a diameter of 140mm and a height of 75 mm was used to hold fresh mortar. The test needle with a cross-section area of 30 mm^2^ (Ap) pressed into the internal 25 mm of the mortar within 10s. The test was started from 1 hour after molding, every 15 min frequency. Then, after the resistance reached 0.3 MPa, it was tested every 10 min frequency, until 4 MPa, one test per minute to 0.5 Mpa. The pressure gauge with a precision of 0.5 MPa was used to record the resistance as NP. The penetration resistance was recorded as fp, fp, and is calculated as shown in Equation (5).

(5) $$f_{p}=\frac{\mathrm{Np}}{\mathrm{Ap}}$$

##### Isothermal Calorimetry

The effects of CHPs worked on the hydration process of fresh mortar were carried out by TAM Air (Waters, Milford, MA, USA). This test was conducted according to GB/T 12959-2008 [36] and operated in a temperature humidity chamber with 100% humidity at 20 °C. The temperature was recorded for the first 48 h at a frequency of once a minute. In this study, the content of CHPs was set at a constant value of 20%, and the types and sizes of CHPs were mainly investigated and discussed.

#### 2.3.3. Mechanical Test

##### Compressive Strength 

The compressive strength was measured according to JGJ70-2009 [37]. Cement specimens were prepared at a size of 70.7 mm × 70.7 mm × 70.7 mm, then cured in a standard curing room at a temperature of 20 ± 1 °C and relative humidity >95%. The compressive strength testing was carried out at a loading rate of 2400 N/s for the specimens at the ages of 7, 28 and 56 days. The strength was calculated from the average of three specimens for each group, and precisely to 0.1 MPa.

##### Flexural Strength

The flexural strength of specimens, denoted by F_max_, was measured by the four-point bending test according to ASTM C348 [38]. The specimens with a size of 40 mm × 40 mm × 160 mm were prepared and cured, then the four-point bending tests were carried out at the age of 28 days. The loading processes were conducted in two steps. Firstly, specimens were loaded at a rate of 5 N/s to 10 N, then the machine was operated by displacement control at the speed of 0.08 mm/min until it broke. The flexural strength was calculated on an average of three specimens per group.

##### Healing Rate of Flexural Strength

After conducting the test of flexural strength, the specimens that were prepared for the healing tests were pre-damaged to 60% F_max_ under the same loading conditions. Then, they were cured in saturated lime water at 23 ± 2 °C for 7, 28 and 56 days, respectively. The secondary flexural strength was measured while the force position remained. Finally, the strength healing ratio was calculated though three specimens per group.

#### 2.3.4. Acoustic Emission Test

##### Active AE

**Figure 5:**
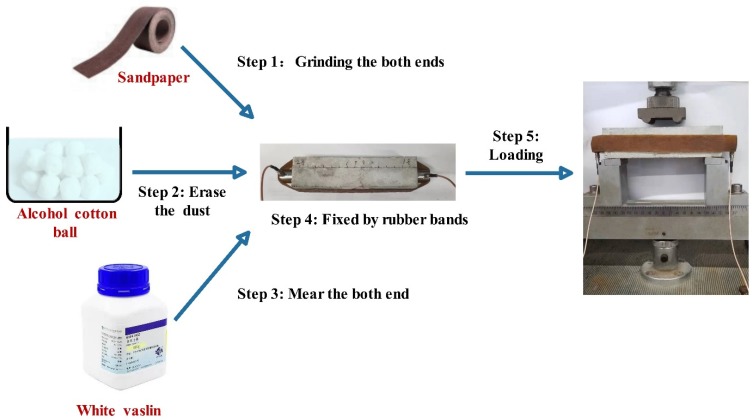
The flow chart of healing test.

The velocity of the longitudinal stress wave through specimens is determined by the transmitting time. The device of AE is shown in Figure 5. The initial transmission time was tested after curing but before the loading of specimens, and it was used to investigate the compactness of mortar, which was recorded as T_0_. The second transmission time was tested after preloading with the position of sensors unmoved, which was recorded as T_1_. After curing in saturated lime water for 7, 28 and 56 days, respectively, the time that pulse passed through the specimens was recorded as T_2_. The damage parameter (D) was estimated by Equations (6) and (7); the damage parameter after loading was D_f_, and the damage parameter after healing was D_h_ [39,40,41]. When the damage parameter decreased to a negative value (D < 0), a higher pulse velocity than that of the intact specimens was indicated.

(6) $$D_{f}=1-{\left(\frac{T_{0}}{T_{1}}\right)}^{2}$$

(7) $$D_{h}=1-{\left(\frac{T_{0}}{T_{2}}\right)}^{2}$$

##### Passive AE 

Caused by the degree of damage, the acoustic emission signal exhibits different energy, amplitude, etc. Real-time dynamic monitoring detects the internal damage of the mortar under stress, and receives these signals by broadband transducers installed at either end of specimens as shown in Figure 5 [41,42]. The transducers (R15) with a broadband response ranging from 60 to 400 kHz and a maximum sensitivity at 150 kHz were used. The cumulative energy of signals was calculated when the load of the four-point bending test reached 60% F_max_ during the preloading and breaking process. The cumulative energy ratio was recorded as the cumulative energy of the secondary load divided by that of the first load. 

## 3. Results and Discussion

### 3.1. Characterization of CHPs

#### 3.1.1. Size Grading and Bulk Density

**Figure 6:**
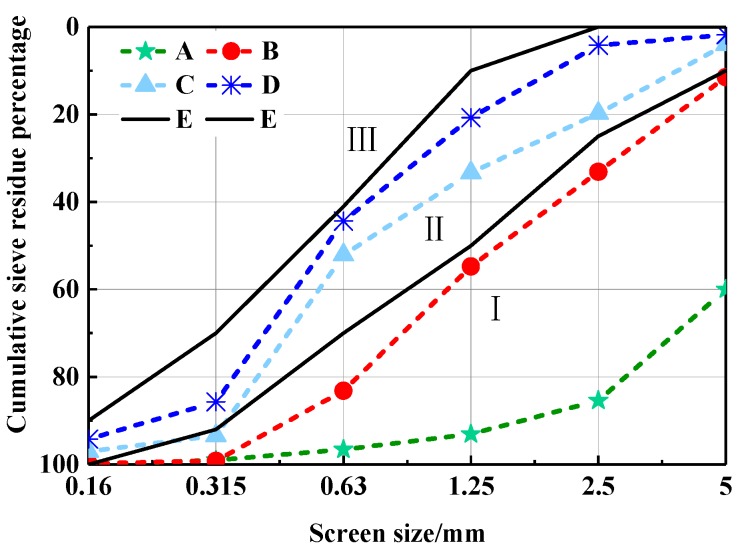
Gradation zone curve of CHPs. (E) Boundary line of the sand in Zone II.

The grain distributed curve of CHPs is shown in Figure 6. It finds that Na_2_CO_3_ decreased the size range of pellets effectively. With the dosages of Na_2_CO_3_ increasing, CHPs are gradually closing to Zone III (fine sand) from Zone I. The changes in grain size of CHPs were caused by the procoagulant effect of Na_2_CO_3_ on cement mortar. The introduced Na_2_CO_3_ hardened the cement paste in the process of mixing and promoted the precipitation of gyrolite, calcite and conversion of calcium silicate gel [23,24]. With the dosages of Na_2_CO_3_ increasing, it is easier for the hardened cement paste to break up during mixing. However, the new surfaces were supplemented with Na_2_CO_3_ or CaCO_3_, which made the dispersed pellets difficult to be consolidated. The main size range of pellets was from 0.35 to 5 mm.

The bulk densities of CHPs with different size ranges are shown in Table 4. It shows that the bulk densities of different size ranges of pellets were similar and the bulk density of the river sand was 1.577 g/cm^3^. Therefore, the bulk densities of CHPs were similar to the Ordinary Portland Cement but 25% lower than river sand. Thus, the introduction of CHPs decreased the densities of specimens.

#### 3.1.2. Microstructural Tests

**Figure 7:**
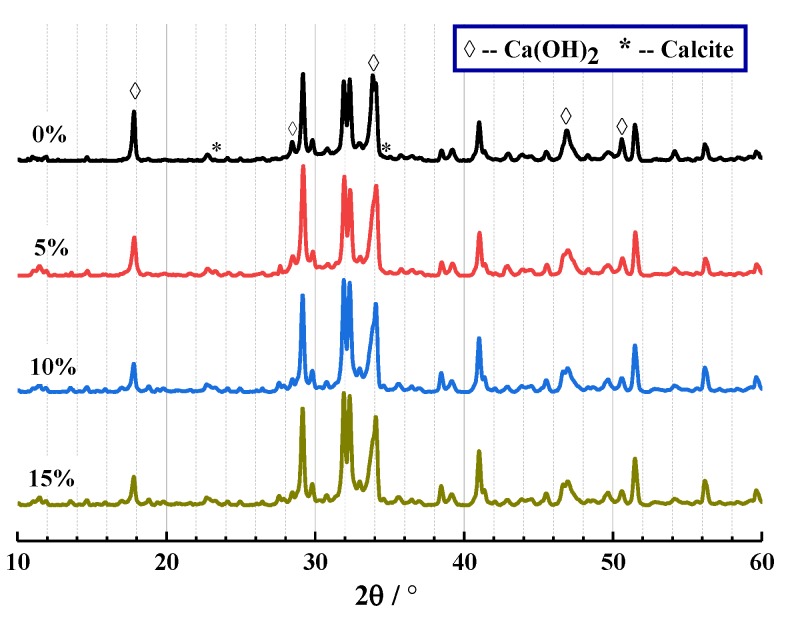
XRD analysis of CHPs.

**Figure 8:**
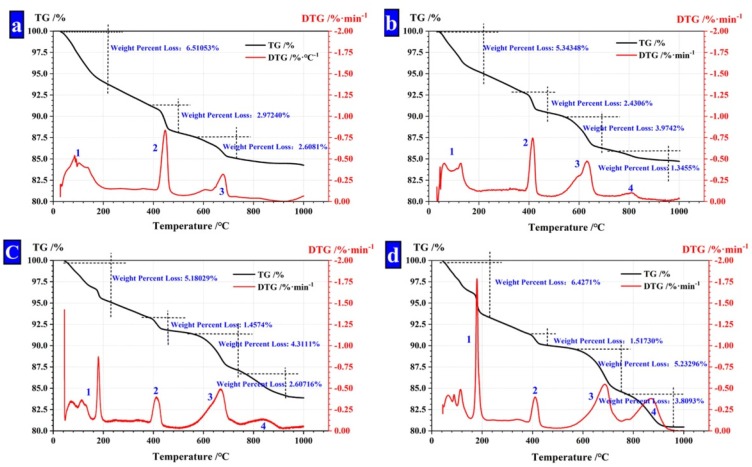
TG analysis of CHPs. (**a**) CHPs with 0% NaCO_3_; (**b**) CHPs with 5% NaCO_3_; (**c**) CHPs with 10% NaCO_3_; (**d**) CHPs with 15% NaCO_3_.

The results of XRD and TG are shown in Figure 7 and Figure 8 below. From Figure 7, the crystal components of CHPs include calcite and Ca(OH)_2_. This proves that Na_2_CO_3_ promoted the production of calcite, which was similar to how the process of carbon dioxide acted. It confirms that the “build-in” carbonation of concrete could be realized by introducing Na_2_CO_3_ internally.

The thermal loss peak between 750 °C and 950 °C (Peak 4) was caused by the thermal decomposition of CaCO_3_. The mass fraction of unreacted Na_2_CO_3._ was calculated as shown in Table 5. It is found that with the dosages of Na_2_CO_3_ increasing, the concentration of unreacted CO_3_^2−^ in CHPs increased, too. With the dosages of Na_2_CO_3_ doubling or increasing three-fold in pellets, the mass fraction of unreacted Na_2_CO_3_ also double or increase three-fold. It means that the concentration of Na_2_CO_3_ in CHPs could be controlled by the dosages of Na_2_CO_3_ introduced.

**Figure 9:**
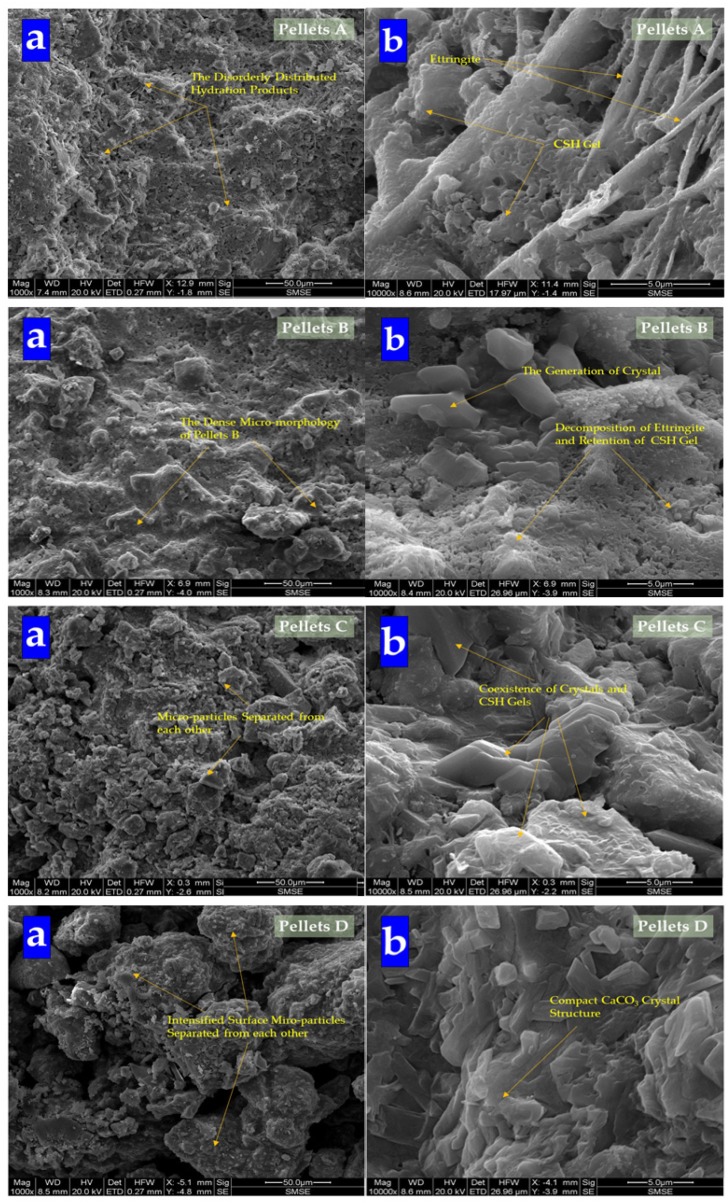
SEM analysis of CHPs. (**a**) Morphology at 50 μm; (**b**) morphology at 5 μm.

The morphology of pellets is shown in Figure 9. It shows that with the dosages of Na_2_CO_3_ increasing, pellets become loose gradually. It affirmed that the addition of Na_2_CO_3_ decreased the mechanical property of cement specimens [43]. While the surface of Pellets A was composed of C-S-H gel and ettringite, with the dosages of Na_2_CO_3_ in pellets increasing, the content of ettringite decreased obviously. Then, the content of gel was also replaced by crystal until the surface of CHPs was gradually covered by crystals. Especially Pellets D, the surface was filled with crystals without gel and ettringite, which means Na_2_CO_3_ promoted the formation of calcite to cross-linking in the cement mortar. The introduction of Na_2_CO_3_ inhibited or consumed the production of ettringite, and promoted the formation of calcite. 

#### 3.1.3. The Ion Release Rate of CHPs

**Figure 10:**
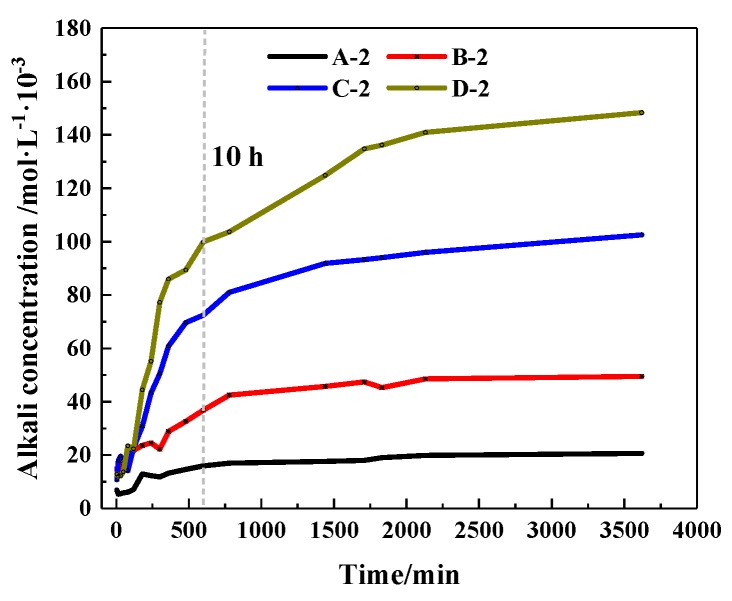
Release rate of alkaline ions under different doses of Na_2_CO_3._

**Figure 11:**
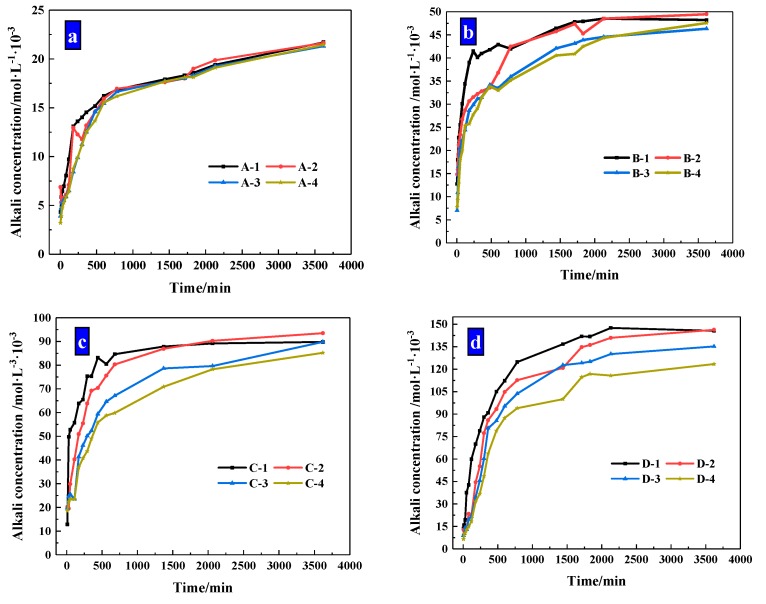
Release Rate of alkaline particles under different sizes. (**a**) Size range of 0.315–0.63 mm; (**b**) size range of 0.63–1.25 mm; (**c**) size range of 1.25–2.5 mm; (**d**) size range of 2.5–5 mm.

The concentration changes of alkaline ions that CHPs dissolved in deionized water are shown in Figure 10. It shows that when soaked for 10 hours, the alkaline concentrations of the leaching solutions of pellets A, B, C and D were, respectively, 77.16%, 74.4%, 70.68% and 68.23% of the final concentration (after soaking for 60 h). When the alkali concentration was 80% of the final concentration, the soaking time of pellets A, B, C and D was 10 h, 12 h, 13 h and 16 h, respectively. It means that with the dosages of Na_2_CO_3_ increasing, the ion release speed was increased while the ion release ratio (ratio of current alkali concentration to final alkali concentration) was decreased. The ion release ratio showed an obvious differentiation for pellets with different size ranges as shown in Figure 11a–d, which illustrates the release rates of CHPs with 0%, 5%, 10% and 15% dosages of Na_2_CO_3_, respectively. Larger pellets put up a lower ion release ratio in deionized water, and this effect was more obvious with the dosages of Na_2_CO_3_ in the pellets increasing. It illustrates that large pellets have a better effect on the storage of CO_3_^2−^ than small pellets, especially in pellets with higher dosages of Na_2_CO_3._ Their denser surface reduced the ion release of CHPs.

### 3.2. The Workability of Mortar with CHPs

#### 3.2.1. Fluidity and setting Time of Fresh Mortar

**Figure 12:**
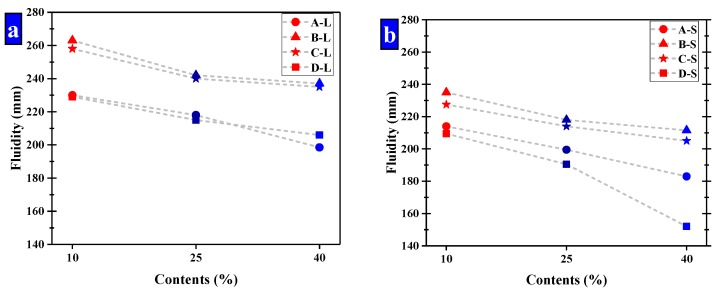
The fluidity of mortar that have been compound allocation. (**a**) Large-sized pellets; (**b**) small-sized pellets.

The size ranges of the pellets were optimized by the fluidity of mortar. While the small pellets have a significant effect of reducing fluidity to be used, two ranges of pellets were selected for further study, namely large and small pellets, respectively. In the group of small pellets, the size ranges of these pellets were mainly 0.65–1.25 mm and 1.25–2.5 mm, which had a ratio of 2. The pellets in the large group were in the ranges of 2.5–5 mm and 1.25–2.5 mm with a ratio of 2. The fluidity of cement mortar is shown in Figure 12 and the fluidity of Group K was 236 mm.

It suggests CHPs decreased the fluidity of mortar obviously compared with Group K except for the large pellets B-L and C-L. It means that the pellets without Na_2_CO_3_ decreased the fluidity of mortar obviously, while Pellets B improved the fluidity of mortar with the low dosage of Na_2_CO_3_ but a smooth surface. The pellets with higher dosages of Na_2_CO_3_ decreased the fluidity of cement mortar obviously, which was caused by the coagulation-promoting effect of Na_2_CO_3_ on the fresh mortar. As such, the fluidity of mortar with Pellets D-S was 32% lower than with Pellets B-S.

The increasing contents of CHPs decreased the fluidity of fresh mortar. When the size range of pellets was 2.5–5 mm, the fluidity was greatly decreased with the contents of pellets increasing. As the contents of pellets increased, the partially hardened regions created in the mortar were interconnected, which decreased in the fluidity of fresh mortar.

The increasing sizes of CHPs improved the fluidity of cement mortar. The fluidity of mortar was higher than 180 mm except for the D-s-3 group, and the fluidity of mortar with large pellets was higher than Group K. It was caused by the fact that the specific surface areas of large pellets were smaller than the river sand, and the contact areas with cement mortar were smaller, which improved the fluidity of mortar.

**Figure 13:**
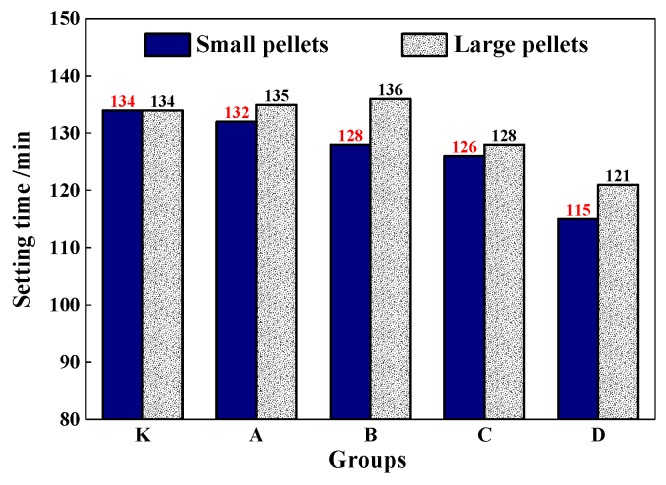
Setting time of mortar mixed with pellets.

The setting time of the mortar was shown in Figure 13. It shows that CHPs had less effect on the setting time of mortar, especially for the large pellets. With the dosages of Na_2_CO_3_ increasing in pellets, the setting time of mortar decreased accordingly. The setting time of mortar with pellets D-S was 15% lower than with pellets B-S. As for the small pellets, when the dosages of Na_2_CO_3_ were 0%, 5%, 10% and 15%, the setting time was reduced by 2 min,6 min, 8 min and 19 min respectively. Combining with the fluidity test, it indicated that the addition of CHPs into fresh cement led to the early rapid release of Na_2_CO_3_. With the setting and hardening of mortar, the release was suppressed and the setting time was partially compensated, especially for Pellets C. This improved the fluidity of mortar and had nearly no reduction effect on the setting time.

#### 3.2.2. Isothermal Calorimetry

**Figure 14:**
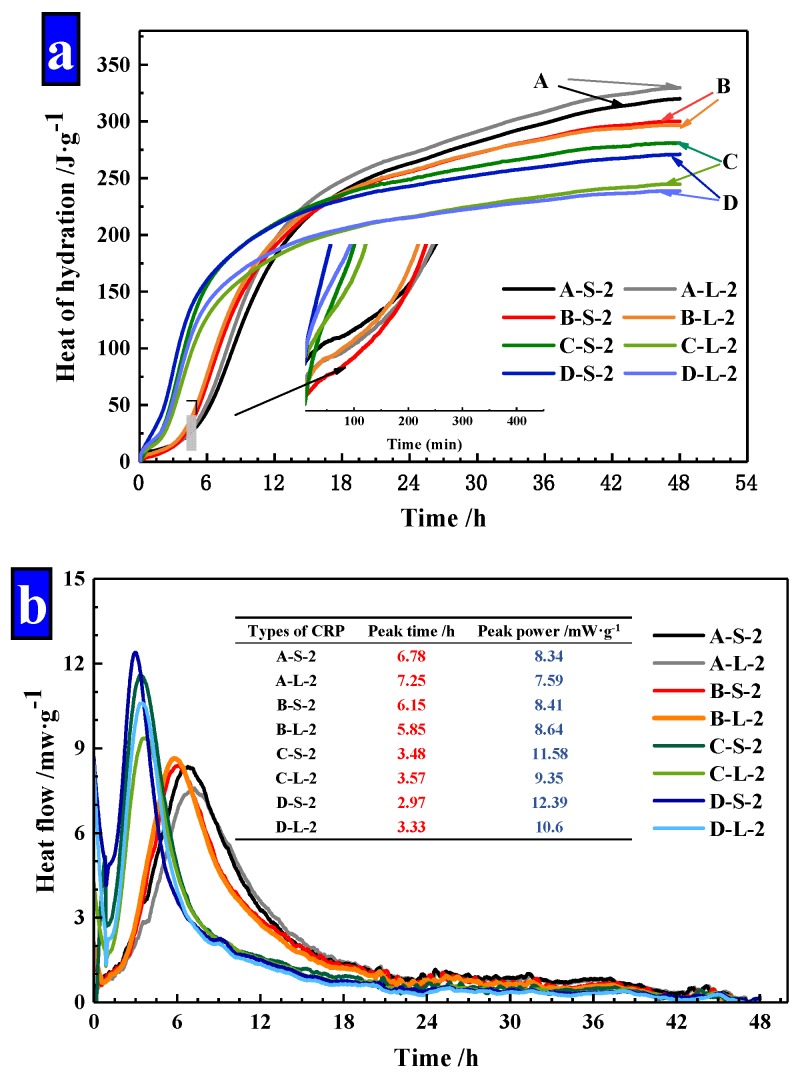
The power and cumulative energy produced per gram of cement for the first 48 h. (**a**) Hydration heat of cement mortar; (**b**) heat flow of cement mortar.

As Figure 14 illustrated, the power and cumulative energy produced per gram of cement for the first 48 h were exhibited. As shown in Figure 14a, it finds that the increased dosages of Na_2_CO_3_ in pellets, decreased the hydration heat of cement mortar obviously and the increased heat release of mortar was mainly in the first 12 h. The hydration heat was gradually slowed down in later stages, which was lower than Group A. This confirms that the release of the material in CHPs can be controlled at the early stage. At the same time, the size ranges of Pellets A and B have less effect on the hydration heat of mortar, but the large pellets (Pellets C and D) decreased the hydration heat of mortar obviously. It indicates that the large pellets (Pellets C and D) have a slow-release effect at the early stage, which achieved the carry and protect of healing materials.

In addition, peak time and peak power values for mixtures were summarized in Figure 14b. The addition of Pellets B showed a slight variation compared with Pellets A. The peak time was shortened to 50% by the addition of Pellets C and D compared with Pellets A. Similarly, the peak power was only slightly affected by the addition of Pellets B, while the addition of Pellets C and D increased the peak power in the range of 38% and 50%. The peak time and peak power of mortar confirmed that CHPs had an efficiency-promoting effect on the hydration reaction of mortar. Pellets A and B had similar peaking time and peak power values, which indicates that the concentration of Na_2_CO_3_ stored in Pellets B was low, while Pellets C and D had a higher concentration of Na_2_CO_3_.

The focus of the present study was to prepare and characterize the new self-healing elements based on the proposed mechanism of “build-in” carbonation. Thus, the varying healing pellets were firstly prepared and investigated, by which Group C exhibited comparatively better performance. Therefore, the strength tests and AE tests of the mortars incorporating Group C were conducted as an example to reveal the influence of the healing element on the mortar as well as its healing effect. The contents and grain sizes of CHPs were mainly discussed in this paper, and the healing effects of specimens were evaluated by comparison with Group K.

### 3.3. The Mechanical Property of Specimens with CHPs

#### 3.3.1. Compressive Strength 

**Figure 15:**
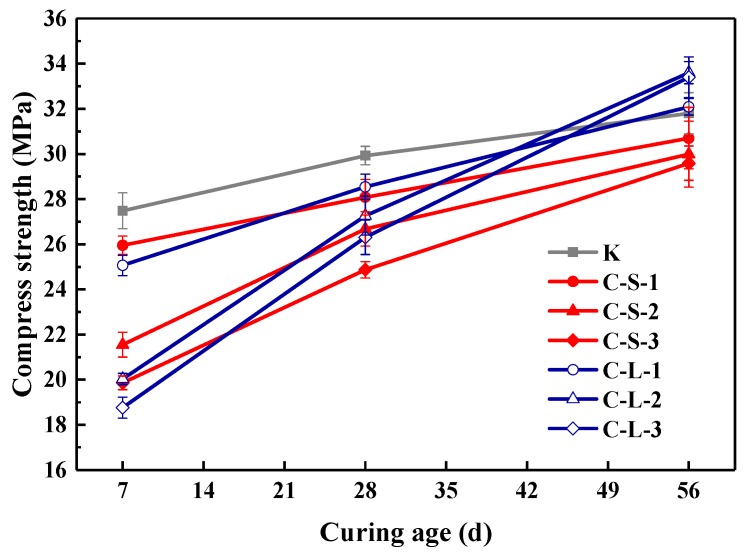
Compressive strength of mortar. Error bars indicate relative standard uncertainty in experimental measurements.

The compressive strength of specimens at the 7th, 28th and 56th day is presented in Figure 15. The addition of large CHPs decreased the early compressive strength but increased the later strength of mortar. On the seventh day, the strength of specimens introduced with CHPs was lower than Group K; meanwhile, the strength of specimens with large pellets was lower than with small pellets. This is caused by the release of Na_2_CO_3_ of large pellets in the early stage being less than that of small pellets. On the 28th day, the strength of specimens with CHPs was lower than Group K, but those with large pellets had higher strength than those with small pellets. On the 56th day, the specimens with large pellets showed the highest strength; the strength of mortar with small pellets was still lower than Group B. This is due to the slow release of CO_3_^2−^ in the healing pellets, which gradually improved the strength of specimens. It showed that the replacement of sand with CHPs increased the strength growth rates of mortar than Group K. In summary, the addition of CHPs into mortar realized the compatibility between healing pellets and cement mortar preliminary, however, the release of Na_2_CO_3_ in mortar challenged the later healing effect.

#### 3.3.2. Flexural Strengths

**Figure 16:**
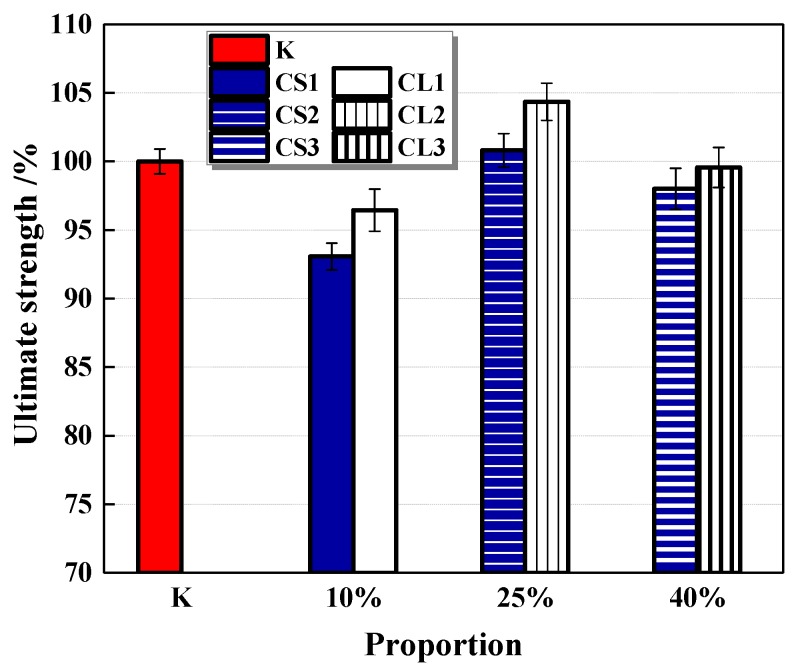
Flexural strength of cement mortar. Error bars indicate relative standard uncertainty in experimental measurements.

The flexural strength of mortar cured for 28 days is shown in Figure 16. It shows that small pellets have a more obvious effect on decreasing the flexural strength of the mortar, which is similar to the trend of compressive strength. Nevertheless, with the contents of CHPs increasing, the trend of strength growth fluctuated. It was caused by the two-blade function of CHPs on the strength growth of mortar. When the content of pellets was 25%, the mortar had higher flexural strengths than Group K, which indicates that the contents of pellets mixed into mortar could be optimized, and the 25% contents of pellets were suitable for the curing age of 28 days.

#### 3.3.3. The Healing Rate of Flexural Strength 

**Figure 17:**
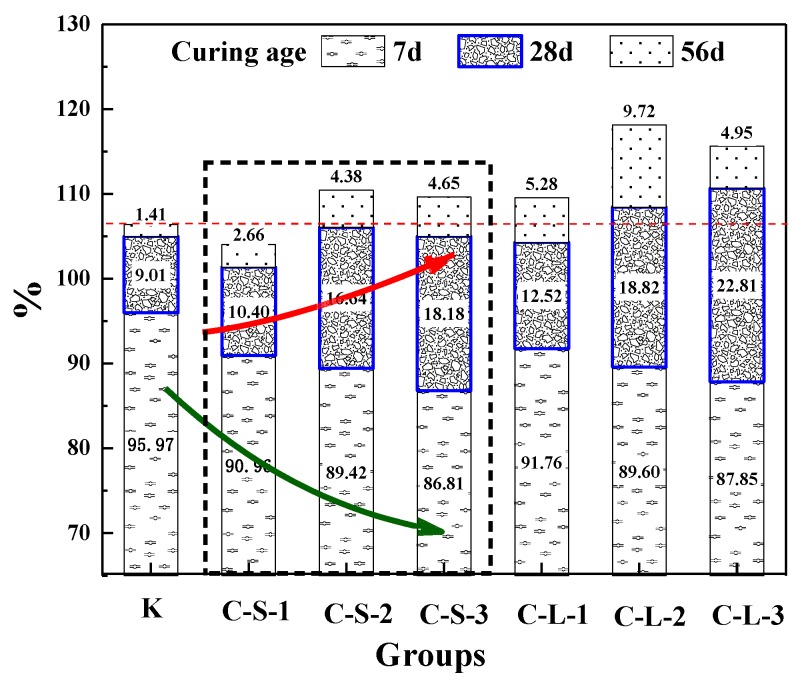
Healing rate of flexural strength.

The healing rate of the flexural strength of mortar is shown in Figure 17. For the curing age of seven days, the healing rate of Group K was better than groups introduced with CHPs, and with the contents of CHPs increasing, the healing rates showed a downward trend. This meant that the hydration of cement in Group K brought a higher healing effect [44,45,46,47]. It was caused by CHPs accelerating the hydration of mortar and improving the cement hydration rate. For the cement used in this experiment, which had an average particle size distribution of 16.1458 μm, the degree of hydration was less than 80% at the age of 28 days under the w/c of 0.5 [47]. Thus, the early healing effects of groups introduced with CHPs were lower than Group K.

For the curing age of 28 days, the growth of flexural healing rates of groups introduced with CHPs was higher than Group K, and it was increased with the contents of CHPs increasing. It is known that the healing effects of CHPs on the pretreatment-damaged specimens were obviously during this period. The groups introduced with large CHPs had higher strength healing rates than those with small CHPs. It means that CHPs can heal the damaged mortar and larger pellets have a more significant self-healing effect. For the curing age of 56 days, the healing rates of mortar were significantly slowed. The strength recovery rate of Group K was lower than groups with CHPs in this stage, which meant that the later healing effects of specimens were improved by CHPs. The strength healing rate of groups C-L-2 was 11.75% higher than Group K. As the ion released rate shows, mortar with large sizes showed better healing effects than those with small pellets, which was caused by the stored Na_2_CO_3_ in the pellets. It means that the addition of CHPs into mortar maintained the healing work well in the later stage.

### 3.4. Evaluation of Healing effect

#### 3.4.1. Active AE

**Figure 18:**
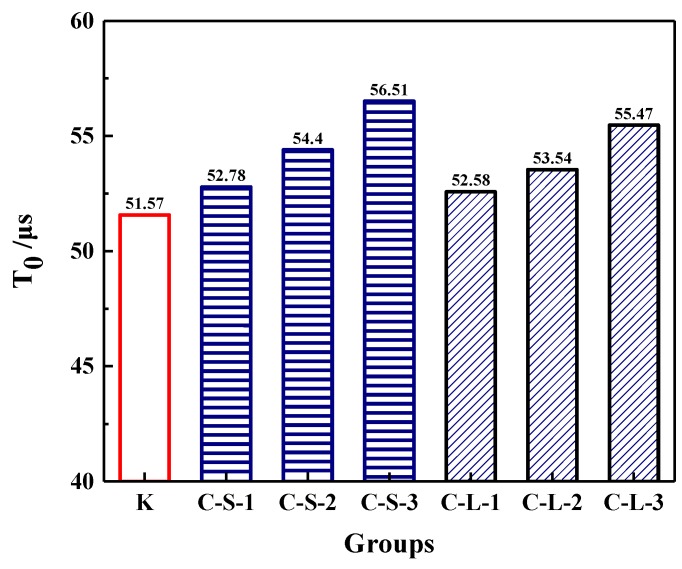
Pulse time of mortar preloading.

**Figure 19:**
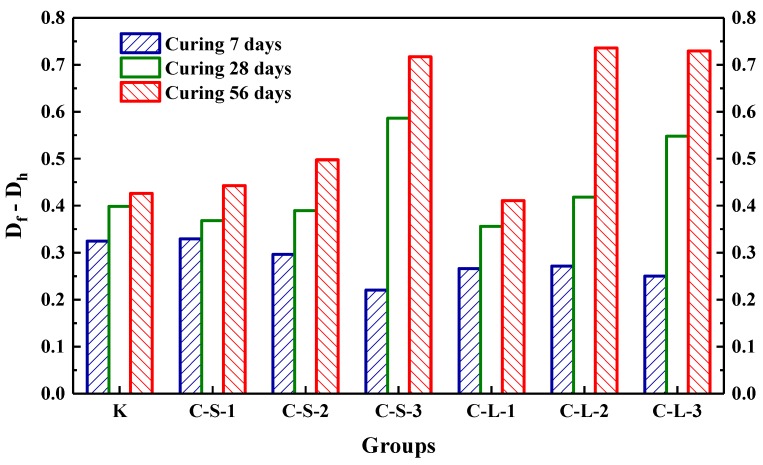
The increment of damage parameter at different curing ages.

The initial transmitting time (T_0_) was performed to investigate the compactness of mortar before loading. As shown in Figure 18, the most compact specimens were Group K, and with the contents of CHPs increasing, T_0_ showed an upward trend. It meant that the addition of CHPs decreased the compact of mortar, which was not only caused by the low density of CHPs, but also by the loosened structures around the pellets (caused by Na_2_CO_3_). When the contents of small pellets were 10%, 25% and 40%, the transmitting time was 2.345%, 5.49% and 9.589% higher than Group K, respectively. Plus, the time that the pulse transmitted through specimens with large pellets was shorter than those with small pellets, meaning that smaller pellets had higher dosages of Na_2_CO_3_ released in mortar, which decreased the density and strength of mortar. T_1_ and T_2_ were tested after specimens had been preloaded and cured for 7, 28 and 56 days, separately, as Figure 19 illustrates. The damage parameter (Df-Dh) showed that CHPs had a long-term filling effect in mortar. For the specimens cured for seven days, it was found that the higher contents of pellets in mortar caused more serious damage after preloading. For the specimens cured for 28 days, the healing effects of mortar with pellets were obvious. When the contents of pellets were 45%, the healing effects of mortar were the best. For the specimens cured for 56 days, it was known that the healing effect of Group K and Group C-S-1 was slightly different after curing for 28 days and 56 days, but the healing effect with small pellets decreased gradually with the increased contents of CHPs, which indicated that the healing effects of mortar with small pellets were not as good as those of large pellets.

#### 3.4.2. Passive AE

**Figure 20:**
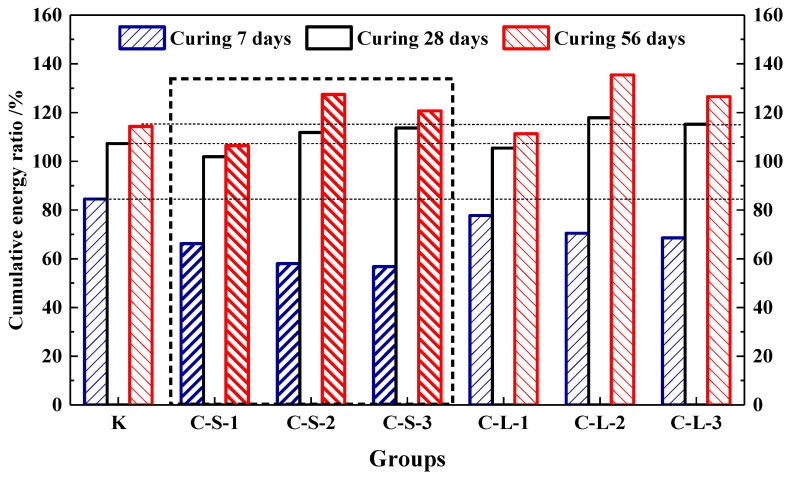
Cumulative energy ratio of mortar.

In this study, the energy produced by the loading process to 60% F_max_ was accumulated. The energy ratio is shown in Figure 20. For the first seven days, Group K showed the highest energy recovery ratio, which was the highest flexural strength healing rate, too. With the contents of pellets increasing in mortar, the energy recovery ratios were gradually decreased. It means that the addition of CHPs decreased the early healing ability of mortar. For the curing age of 28 days, there was an obvious increase in the energy recovery ratios of the mortar with CHPs, which meant that pellets had a good healing efficiency on concrete than Group K in this period. The energy recovery ratios were gradually increased with the contents of pellets increasing. It means that the addition of CHPs increased the bonding strength of mortar, which produced higher energy during loading. For the curing age of 56 days, the increase in energy recovery ratios was a little less. The groups introduced with CHPs had a better healing effect than Group K. Groups C-S-2 and C-L-2 had the highest energy recovery ratio, while the 40% contents of CHPs had a worse energy recovery ratio. It could be concluded that the flexural strength healing ratios of mortar were improved by CHPs. When the content of CHPs was 25%, the highest flexural strength of the mortar was after healing.

## 4. Conclusions

The introduction of new self-healing pellets (CHPs) realized the “build-in” carbonation of mortar. CHPs present a circular granular form with a density of 25% lower than river sand, and the addition of Na_2_CO_3_ decreased the size range of pellets, promoting the production of calcite. With the dosages of Na_2_CO_3_ increasing, the content of calcite increased evidently to cross-linking, and the large pellets had a more obvious ability to reduce the release of ions in water. 

Pellets decreased the fluidity of the mortar except for pellets B-L and C-L. Increasing contents or decreasing the size ranges of pellets also decreased the fluidity of mortar. CHPs had less effect on the setting time of mortar, and with the dosages of Na_2_CO_3_ increasing in pellets, the hydration heat of cement mortar decreased obviously. This means that CHPs reduced the early release of Na_2_CO_3_ in fresh mortar.

The compatibility between healing pellets and mortar was basically realized. The addition of CHPs reduced the early compressive strength of mortar, but the later strength gradually caught up with or exceeded the control group. The larger pellets brought a higher later strength of mortar than small pellets.

The healing effect of CHPs in mortar is extended. With the increase of contents or size ranges of pellets, the microstructure of mortar was much looser. The mortar with CHPs had an obvious filling effect and strength healing rate than Group K. While cured for 28 or 56 days, there was still showed an obvious filling effect on cement mortar.

## Figures and Tables

**Table 1:** Chemical composition of raw materials (wt %).

Materials	CaO	SiO_2_	Al_2_O_3_	Fe_2_O_3_	SO_3_	K_2_O	MgO	TiO_2_	Cl
CEM	65	20.90	4.56	3.23	2.65	0.87	0.65	0.22	0.05
Quartz sand	0.05	99.27	0.3	0.03	-	0.1	0.05	-	-

**Table 2:** Mix proportion of cement-based healing pellets (CHPs).

Types	$\frac{Na_{2}CO_{3}}{CEM}/\%$	CEM/g	Water/g	Na_2_CO_3_/g	Quartz Sand/g
A	0%	100	22	0	30
B	5%	100	22	5	30
C	10%	100	22	10	30
D	15%	100	22	15	30

**Table 3:** Mixing ratio of cement mortar.

Groups	CHPs	Size	$\frac{CHPs}{River\;Sand}\;/\%\;$	CEM/g	Water/g	River Sand/g
K	-	-	0	500	250	1250
A-L-1	A	Large	10			1125
A-L-2		Large	25			937.5
A-L-3		Large	40			750
A-S-1		small	10			1125
A-S-2		small	25			937.5
A-S-3		small	40			750
B-L-1	B	Large	10			1125
B-L-2		Large	25			937.5
B-L-3		Large	40			750
B-S-1		small	10			1125
B-S-2		small	25			937.5
B-S-3		small	40			750
C-L-1	C	Large	10			1125
C-L-2		Large	25			937.5
C-L-3		Large	40			750
C-S-1		small	10			1125
C-S-2		small	25			937.5
C-S-3		small	40			750
D-L-1	D	Large	10			1125
D-L-2		Large	25			937.5
D-L-3		Large	40			750
D-S-1		small	10			1125
D-S-2		small	25			937.5
D-S-3		small	40			750

**Table 4:** Bulk densities of CHPs.

Size/mm	0.315–0.63	0.63–1.25	1.25–2.5	2.5–5
Bulk density/g·cm^-3^	1.166	1.133	1.124	1.128

**Table 5:** The mass fraction of unreacted Na_2_CO_3._

Types of CHPs	Mass Fraction/%
A	0
B	0.5966
C	1.2968
D	1.7748
